# Gabapentinoids for oncologic pruritus: a retrospective cohort study

**DOI:** 10.1093/oncolo/oyag431

**Published:** 2026-10-28

**Authors:** Alim Osman, Ian Nykaza, Sarah L Spaulding, Stephen W Dusza, Lavanya Mittal, Allison Gordon, Shamir Geller, Cesar A Virgen, Veronica Rotemberg, Doris M Ponce, Jasmeet C Singh, Jodi Victoria Mones, Alina Markova

**Affiliations:** Dermatology Service, Department of Medicine, Memorial Sloan Kettering Cancer Center, New York, NY 10021, United States; Dermatology Service, Department of Medicine, Memorial Sloan Kettering Cancer Center, New York, NY 10021, United States; Weill Cornell Medical College, New York, NY 10065, United States; Dermatology Service, Department of Medicine, Memorial Sloan Kettering Cancer Center, New York, NY 10021, United States; Dermatology Service, Department of Medicine, Memorial Sloan Kettering Cancer Center, New York, NY 10021, United States; Dermatology Service, Department of Medicine, Memorial Sloan Kettering Cancer Center, New York, NY 10021, United States; Weill Cornell Medical College, New York, NY 10065, United States; Dermatology Service, Department of Medicine, Memorial Sloan Kettering Cancer Center, New York, NY 10021, United States; Weill Cornell Medical College, New York, NY 10065, United States; Dermatology Service, Department of Medicine, Memorial Sloan Kettering Cancer Center, New York, NY 10021, United States; Weill Cornell Medical College, New York, NY 10065, United States; Dermatology Service, Department of Medicine, Memorial Sloan Kettering Cancer Center, New York, NY 10021, United States; Weill Cornell Medical College, New York, NY 10065, United States; Dermatology Service, Department of Medicine, Memorial Sloan Kettering Cancer Center, New York, NY 10021, United States; Weill Cornell Medical College, New York, NY 10065, United States; Weill Cornell Medical College, New York, NY 10065, United States; Bone Marrow Transplant Service, Department of Medicine, Memorial Sloan Kettering Cancer Center, New York, NY 10021, United States; Breast Medicine Service, Department of Medicine, Memorial Sloan Kettering Cancer Center, New York, NY 10021, United States; Hematology Service, Department of Medicine, Memorial Sloan Kettering Cancer Center, New York, NY 10021, United States; Dermatology Service, Department of Medicine, Memorial Sloan Kettering Cancer Center, New York, NY 10021, United States; Weill Cornell Medical College, New York, NY 10065, United States

**Keywords:** pzruritus, itch, cancer, gabapentinoids, gabapentin, pregabalin

## Abstract

**Background:**

Pruritus is a debilitating symptom in oncologic patients which often leads to treatment interruptions or dose reductions and reduced quality of life. Gabapentinoids such as gabapentin and pregabalin approved for postherpetic neuralgia, partial seizures, neuropathic pain, and fibromyalgia have also demonstrated efficacy in treating non-oncologic forms of pruritus. We report on the safety and efficacy of gabapentinoids for oncologic pruritus.

**Patients and methods:**

This was a single-center retrospective cohort study conducted at the Memorial Sloan Kettering Cancer Center (MSK). All patients from January 04, 2019 to January 8, 2024 prescribed gabapentinoids for oncologic pruritus were retrospectively identified.

**Results:**

224 patients were included; the average age of the cohort was 63.2 ± 15 years and were mostly male (54%). Patients were treated for pruritus secondary to drug-induced causes (60.3%), malignancy/paraneoplastic (15.2%), cutaneous graft-versus-host disease (13.4%), and other etiologies (11.2%). Pregabalin was more frequently prescribed than gabapentin (80.8% vs. 19.2%). Ninety percent of patients (*n* = 202) experienced ≥1 grade improvement in CTCAE grade, at a median dosage 50 mg and 300 mg for pregabalin and gabapentin, respectively. The median time to patient-reported initial symptom improvement was 18.5 days. The median time to the documented ≥1 CTCAE grade improvement was 3.5 months (95% CI, 3.0-4.7), with no significant difference observed between pruritus etiologies. Adverse events were reported in 14.7% of patients, most commonly sedation (8.5%). Only three patients discontinued gabapentinoids due to adverse events.

**Conclusion:**

Our results demonstrate that gabapentinoids are a safe and effective way to treat oncologic pruritus.

Implications for practice Pruritus is a common, debilitating symptom among patients undergoing cancer treatment. First-line treatments include topical corticosteroids, topical pramoxine, and oral antihistamines, which may have incomplete efficacy, prompting use of systemic steroids with associated toxicity. More tolerable long-term treatments are needed. In a retrospective cohort of 224 patients treated across various etiologies, 90% achieved at least 1 CTCAE grade improvement at a median daily dose of 50 mg pregabalin and 300 mg gabapentin, with adverse events reported in 14.7% of patients, most commonly sedation. Gabapentinoids represent a safe and effective option for oncologic pruritus.

## Introduction

Pruritus is a debilitating symptom in oncologic patients associated with treatment interruptions or dose reductions and reduced quality of life, most frequently arising from treatment-related toxicities, paraneoplastic processes, and immune dysregulation.^1–3^ Chemotherapy-related pruritus varies widely by agent, with taxanes reporting an overall incidence of 14%.^4^ Targeted therapies such as epidermal growth factor receptor (EGFR) inhibitors are associated with higher frequencies of pruritus, with all-grade incidence exceeding 50% depending on the agent used.^5^ Paraneoplastic pruritus is most commonly associated with hematologic malignancies such as polycythemia vera, affecting 15%-50% of patients.^6^ Pruritus can also occur secondary to cutaneous involvement by the tumor itself, driving eosinophil production and CD4 helper T-cell activation, leading to IL-31 driven sensitization of nerve endings.^2^ Immune-mediated toxicities such as cutaneous graft-versus-host disease (GVHD) have been associated with a prevalence of pruritus ranging from 51% to 64% in acute and chronic cutaneous GVHD, respectively.^7^

Initial management for pruritus involves topical therapies, including emollients, topical corticosteroids, and topical menthol or camphor preparations. Systemic agents such as antihistamines or less commonly antidepressants, such as mirtazapine or sertraline, or anti-emetics like aprepitant, are used when topical interventions are insufficient. Phototherapy may be considered but requires 2-3 in-office treatments weekly. For refractory cases, biologic agents such as dupilumab (targeting IL-4Rα) and omalizumab (targeting IgE) have also been used off label.^8^ Gabapentinoids, such as gabapentin and pregabalin, are approved for the treatment of postherpetic neuralgia, partial seizures, neuropathic pain, and fibromyalgia. These have also effectively treated various forms of pruritus, particularly of uremic or neuropathic origin with limited adverse events including dizziness, somnolence, and peripheral edema.^9^ Their effect is believed to stem primarily from the inhibition of α2δ subunits of voltage-gated calcium channels in the dorsal root ganglion, thereby increasing the threshold required for neuronal excitability, which contributes to itch (Figure 1).^10^^,^^11^

**Figure 1.**
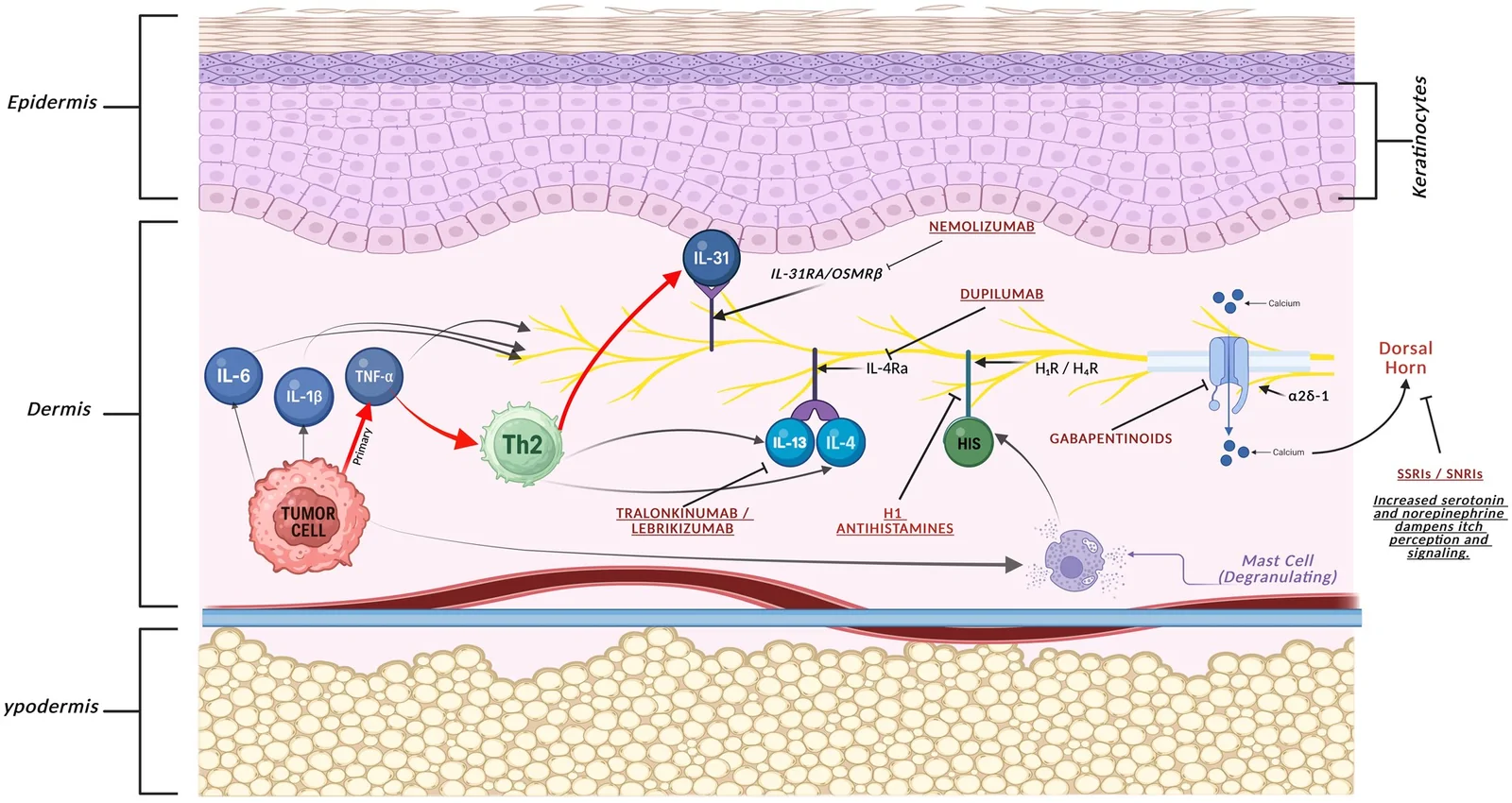
Mechanisms of oncologic pruritus and associated therapeutics. This schematic illustrates pathways of oncologic pruritus signaling. Tumor cells release pro-inflammatory cytokines such as IL-6, IL-1β, and TNF-α, sensitizing nearby nerve endings and stimulating Th2 differentiation and activation.^19^ Activated Th2 cells produce downstream pruritogenic cytokines, primarily IL-31, which binds to the IL-31RA/OSMRβ receptor on peripheral nerve endings,^20^ activating JAK/STAT signaling and resulting in sensitization of the nerve fibers. Nemolizumab inhibits this pathway by targeting the IL-31RA receptor. IL-4 and IL-13 are also produced by Th2 cells, sensitizing the nerve through the IL-4Rα receptor.^17^ Dupilumab blocks IL-4Rα, inhibiting neuronal sensitization mediated by IL-4 and IL-13. Tralokinumab and lebrikizumab inhibit this pathway through selective IL-13 blockade. Tumor cells also directly activate mast cells, causing histamine degranulation and binding to the H1R and H4R receptors.^21^ The cumulative input of all pruritic signaling activates voltage-gated calcium channels in the distal fiber and primarily in the dorsal horn, causing the release of excitatory neurotransmitters such as glutamate and substance P that drive the sensation of itch.^11^ Gabapentinoids exert their anti-pruritic effects by binding to the α2δ-1 subunit of the voltage-gated calcium channels, inhibiting depolarization.^11^ SSRIs/SNRIs may reduce pruritus through central neuromodulation of itch perception and spinal itch signaling.^16^

Despite the safety and efficacy of gabapentinoids in uremic and neuropathic itch, their efficacy and safety for etiologies of oncologic pruritus have not been described. The purpose of our study was to retrospectively evaluate the clinical efficacy and safety of gabapentinoids in oncologic pruritus.

## Methods

### Patient selection/inclusion criteria

This study was approved by the Institutional Review Board at Memorial Sloan Kettering Cancer Center (IRB Protocol 16-458). Informed consent was waived given the retrospective nature of the study. Patients who received gabapentinoids (gabapentin, pregabalin, mirogabalin, or encarabril) at Memorial Sloan Kettering Cancer Center (MSKCC) from January 4, 2019 to January 8, 2024 with ICD code L29 or either “pruritus,” “pruritic,” or “itch” in the clinical notes were identified. Chart review was performed to identify patients who were prescribed and took gabapentinoids for oncologic pruritus. Oncologic pruritus was defined as any pruritus originating from: malignancy, oncologic therapies, paraneoplastic syndromes, or cancer-related immune dysregulation, including conditions such as cutaneous GVHD. Patients were excluded if they were noncompliant with gabapentinoid therapy, were prescribed gabapentin or pregabalin for pain or another non-pruritic indication and did not undergo dose modification for pruritus, had insufficient follow-up or documentation to assess treatment response, or died or transferred care prior to any follow-up assessment after gabapentinoid initiation.

### Pruritus grading

Pruritus was graded using the Common Terminology Criteria for Adverse Events (CTCAE) Version 5.0 for Pruritus.^12^ Documented CTCAE grades were recorded from patient medical records at each visit. When documentation was unavailable, patients were retrospectively graded based on documented symptoms. CTCAE criteria for pruritus are as follows:

- Grade 1: Mild or localized; topical intervention indicated.
- Grade 2: Widespread and intermittent; skin changes from scratching (eg, edema, papulation, excoriations, lichenification, and oozing/crusts); oral intervention indicated; limiting instrumental ADL.
- Grade 3: Widespread and constant; limiting self-care ADL or sleep; systemic corticosteroid or immunosuppressive therapy indicated.

### Statistical analysis

The primary endpoints were defined as follows: the proportion of patients experiencing a ≥ 1 grade improvement in CTCAE pruritus grade from baseline to follow-up, overall and stratified by indication. Secondary endpoints included patient-reported perceptions of pruritus relief, antineoplastic therapy interruption, and adverse events. Descriptive statistics were performed to summarize baseline demographics and clinical characteristics of the patient cohort using frequencies and relative frequencies. Clinical efficacy was assessed by calculating the frequency in reduction by at least 1 CTCAE grade. Exact binomial 95% confidence intervals were estimated for rates of pruritus by etiologic nature. Wilcoxon sign-rank test was used to assess differences in gabapentin dose by patient characteristics. All statistical analyses were performed with Stata/MP v19.

## Results

A total of 224 patients with oncologic pruritus treated with gabapentinoids were included in the analysis (Table 1). The average age of the cohort was 63.2 ± 15 years, with the majority being male (54%) and White (67%).

**Table 1.** Baseline demographics.

Patient demographics	*N* (%); *N* = 224 patients
**Age (years)**	
** Mean (SD)**	63.2 (15)
**Sex, *N* (%)**	
** Female**	103 (46.0)
** Male**	121 (54.0)
**Race, *N* (%)**	
** White**	150 (67.0)
** Black/African American**	32 (14.3)
** Asian**	28 (12.5)
** Other/unknown**	14 (6.3)
**Gabapentinoid, *N* (%)**	
** Pregabalin**	181 (80.8)
** Gabapentin**	42 (19.2)
**Pruritus etiology, *N* (%)**	
** Drug-induced**	135 (60.3)
** Checkpoint inhibitors**	61 (27.2)
** Targeted therapy/small molecules**	34 (15.2)
** Chemotherapy/cytotoxic**	17 (7.6)
** Antibody-drug conjugates**	7 (3.1)
** Antibiotics**	5 (2.2)
** Hormonal/endocrine therapies**	3 (1.3)
** Other**	8 (3.6)
** Malignancy-related/paraneoplastic**	34 (15.2)
** CTCL/mycosis fungoides**	24 (10.7)
** Cutaneous metastasis/malignant wound**	8 (3.6)
** Basal cell carcinoma**	1 (0.4)
** Small lymphocytic lymphoma**	1 (0.4)
** Cutaneous GVHD**	30 (13.4)
** Acute GVHD**	17 (7.6)
** Chronic GVHD**	13 (5.8)
** Other**	25 (11.2)
** Eczematous dermatitis**	7 (3.1)
** Atopic dermatitis**	3 (1.3)
** Bullous pemphigoid**	3 (1.3)
** Eosinophilic folliculitis**	3 (1.3)
** Multifactorial**	2 (0.9)
** Radiation dermatitis**	2 (0.9)
** Dermatomyositis**	1 (0.4)
** Eosinophilic eruption of hematologic malignancy**	1 (0.4)
** Engraftment syndrome**	1 (0.4)
** Lichen planus**	1 (0.4)
** Prurigo nodularis**	1 (0.4)

This table summarizes age, sex, race, gabapentinoid prescribed, and pruritus etiology among 224 patients included in the retrospective cohort. Etiologies are further stratified into drug-induced, malignancy-related/paraneoplastic, cutaneous GVHD, and other causes.

Abbreviations: CTCL, cutaneous T-cell lymphoma; GVHD, graft-versus-host disease; *N*, number; SD, standard deviation.

Drug-induced pruritus (60.3%) was the most frequent etiology of pruritus, followed by malignancy-related/paraneoplastic pruritus (15.2%), cutaneous GVHD (13.4%), and other etiologies (11.2%, Table 1). Among drug-induced pruritus, immune-checkpoint inhibitors were the most frequently associated therapy (45.2%), followed by targeted therapy/small molecules (25.2%), chemotherapy/cytotoxic agents (12.6%), and antibody-drug conjugates (5.2%). Among malignancy-associated or paraneoplastic pruritus, cutaneous lymphoma (CL) was the most common underlying diagnosis (*n* = 24), predominantly consisting of mycosis fungoides, with additional cases of Sézary syndrome, peripheral T-cell lymphoma, and adult T-cell leukemia/lymphoma. Among patients with chronic cutaneous GVHD (*n* = 13), epidermal subtypes (lichen planus-like, papulosquamous, and eczematous) were most common (*n* = 9), followed by sclerotic involvement (*n* = 4). Other etiologies included eczematous dermatitis (*n* = 7), atopic dermatitis (*n* = 3), bullous pemphigoid (*n* = 3), and eosinophilic folliculitis (*n* = 3), among others.

Most patients improved by ≥1 CTCAE grade (90.2%), including drug-induced (91.1%), acute and chronic cutaneous GVHD (90.0%), and malignancy-related/paraneoplastic (88.2%). There was no significant difference in response (*P* = .31) or in time to response (*P* = .15) between the different pruritus etiologies. Of the 174 patients with available patient-reported outcome data, 141 (63.0%) reported experiencing improvement and attributed it to the gabapentinoid. Pregabalin was more frequently prescribed than gabapentin (80.8% vs 19.2%), with median daily doses at improvement of 50 mg and 300 mg, respectively (Table 2). When stratified by age (<70 and ≥70), patients ≥70 years of age achieved improvement at a significantly lower daily dose of pregabalin (50 mg vs. 100 mg, *P* < .001). There was no significant difference in dosage of improvement between age groups in patients taking gabapentin (*P* = .58). Average time to improvement of ≥1 CTCAE grade was 3.5 months. Median patient-reported improvement in symptoms was 18.5 days.

**Table 2.** Gabapentinoid treatment outcomes.

Gabapentinoid treatment outcomes		*N* = 224 patients
**Dose at improvement mg/day, median [IQR] (range)**	Pregabalin	50 [25], (25-300)
	Gabapentin	300 [50], (100-3600)
**Patient reported response, *N* (%)**	Improved	141 (63.0)
	No improvement	33 (14.7)
	Unknown	50 (22.3)
**Pruritus CTCAE grade change, *N* (%)**	Improved ≥ 1 grade	202 (90.2)
	No change	16 (7.1)
	Worsened	6 (2.7)
**Adverse events, *N* (%)**	None reported	191 (85.3)
	Sedation	19 (8.5)
	Headache	2 (0.9)
	Leg swelling	2 (0.9)
	Other	6 (2.7)

This table summarizes median dose at improvement for pregabalin and gabapentin, patient-reported symptomatic response, CTCAE pruritus grade change, and reported adverse events among the full cohort. CTCAE improvement was defined as a reduction of at least 1 pruritus grade from baseline.

Abbreviations: CTCAE, Common Terminology Criteria for Adverse Events; IQR, interquartile range; *N*, number.

Response rates were high across all drug-induced subcategories, including checkpoint inhibitor-associated pruritus (90.2%; 95% CI, 80.2%-95.4%), targeted therapy/small molecules (91.2%; 95% CI, 77.0%-97.0%), and chemotherapy/cytotoxic agents (88.2%; 95% CI, 65.7-96.7%). Among malignancy-related etiologies, patients with CTCL/mycosis fungoides achieved a response rate of 91.7% (95% CI, 74.2-97.7%). Response rates by pruritus etiology are summarized in Table 3. Concurrent use of other anti-pruritic or dermatologic therapies was common including topical corticosteroids (96.9%), antihistamines/anti-allergic agents (60.7%), biologics (31.2%), systemic corticosteroids (25.4%), topical calcineurin inhibitors (16.1%), and JAK inhibitors (7.6%) were among the most frequently used concomitant treatments.

**Table 3.** Gabapentinoid response rates by pruritus etiology.

Pruritus etiology	Responders, *n*	Response rate, % (95% CI)
**Drug-induced (*n* = 135)**	123	91.1% (85.1-94.8)
**Checkpoint inhibitors (*n* = 61)**	55	90.2% (80.2-95.4)
**Targeted therapy/small molecules (*n* = 34)**	31	91.2% (77.0-97.0)
**Chemotherapy/cytotoxic (*n* = 17)**	15	88.2% (65.7-96.7)
**Antibody-drug conjugates (*n* = 7)**	6	85.7% (48.7-97.4)
**Antibiotics (*n* = 5)**	5	100.0% (56.6-100.0)
**Hormonal/endocrine (*n* = 3)**	3	^a^
**Other (*n* = 8)**	8	100.0% (67.6-100.0)
**Malignancy-related/paraneoplastic (*n* = 34)**	30	88.2% (73.4-95.3)
**CTCL/mycosis fungoides (*n* = 24)**	22	91.7% (74.2-97.7)
**Cutaneous metastasis/malignant wound (*n* = 8)**	7	87.5% (52.9-97.8)
**Basal cell carcinoma (*n* = 1)**	0	^a^
**Small lymphocytic lymphoma (*n* = 1)**	1	^a^
**Cutaneous GVHD (*n* = 30)**	27	90.0% (74.4-96.5)
**Acute GVHD (*n* = 17)**	16	94.1% (73.0-99.0)
**Chronic GVHD (*n* = 13)**	11	84.6% (57.8-95.7)
**Other/unspecified (*n* = 25)**	22	88.0% (70.0-95.8)
**Eczematous dermatitis (*n* = 7)**	5	71.4% (35.9-91.8)
**Atopic dermatitis (*n* = 3)**	3	^a^
**Bullous pemphigoid (*n* = 3)**	3	^a^
**Eosinophilic folliculitis (*n* = 3)**	3	^a^
**Multifactorial (*n* = 2)**	2	^a^
**Radiation dermatitis (*n* = 2)**	2	^a^
**Dermatomyositis (*n* = 1)**	0	^a^
**Engraftment syndrome (*n* = 1)**	1	^a^
**Eosinophilic eruption of hematologic malignancy (*n* = 1)**	1	^a^
**Lichen planus (*n* = 1)**	1	^a^
**Prurigo nodularis (*n* = 1)**	1	^a^

This table presents the number of responders and response rates with exact binomial 95% confidence intervals across major pruritus etiologies and subcategories. Response was defined as improvement of at least 1 CTCAE pruritus grade following gabapentinoid treatment.

Abbreviations: CI = confidence interval; CTCL = cutaneous T-cell lymphoma; GVHD = graft-versus-host disease.

a Indicate categories with sample size too small to report, defined as *n* < 5.

The majority of patients tolerated gabapentinoids without adverse events (85.3%). The most frequent adverse events were sedation (8.5%), followed by leg swelling (0.9%), headache (0.9%), and single reported cases of weakness, tremors, mild weight gain, and fatigue. Mean age was similar between patients with and without sedation (60.9 vs 63.5 years), suggesting no clear age-related trend in this cohort. Three patients (1.3%) discontinued gabapentinoid therapy due to an adverse event. One patient on pregabalin developed fatigue and diminished concentration, which resolved after discontinuation. A second patient on pregabalin developed sedation/drowsiness that worsened over time, leading to discontinuation. Resolution was not clearly documented, and the patient had underlying comorbidities (chronic graft-versus-host disease, myositis) that may independently contribute to fatigue. A third patient developed leg swelling attributed to gabapentin. However, additional medical records on this patient’s management could not be verified. Thirteen patients (5.8%) transitioned between gabapentin and pregabalin during their treatment course, although most did not have a documented reason for the switch. Of the switches without a documented clinical reason, 5 of 8 occurred from gabapentin to pregabalin, which may in part reflect third-party payer step-therapy requirements mandating a gabapentin trial prior to pregabalin authorization.

## Discussion

Our findings show that gabapentinoids were effective in improving oncologic pruritus across multiple etiologies including drug-related, malignancy-associated/paraneoplastic, and immune-mediated. Use of gabapentinoids for oncologic pruritus offers an adjunctive treatment to topical therapies and antihistamines, and an alternative to systemic corticosteroids, dupilumab, and omalizumab.^8^

Over 90% of our patients experienced at least a 1 grade improvement in pruritus severity, with less than 15% reporting adverse events. Pregabalin was prescribed more frequently than gabapentin, and pruritus control was achieved at a relatively low median daily dose of 50 mg for pregabalin and 300 mg for gabapentin. While gabapentin and pregabalin have previously been reported to both successfully treat uremic pruritus where renal function is impaired, our institution prefers the use of pregabalin for pruritus due to its rapid rate of absorption and lower variability in bioavailability in the setting of normal renal function when compared to gabapentin^9^^,^^13^ No standardized protocol exists for transitioning between gabapentin and pregabalin. Published approaches include a commonly cited 6:1 dose-equivalency ratio (total daily gabapentin dose divided by 6 to approximate an equivalent pregabalin dose), though this ratio’s derivation has been debated and is complicated by gabapentin’s nonlinear bioavailability.^14^ Pharmacokinetic modeling suggests that both immediate switching and gradual cross-titration achieve comparable steady-state drug exposure within approximately 1 day, though gradual tapering is generally recommended to minimize discontinuation symptoms.^14^ In our cohort, transitions were managed at the treating physician’s discretion, most often as a direct switch.

Current treatment options for oncologic pruritus differ based on etiology. Drug-induced pruritus, particularly immune-checkpoint inhibitor-associated pruritus, which has been reported in up to 30.7% of patients, involves stepwise management as outlined by the National Comprehensive Cancer Network supportive care guidelines.^3^^,^^5^ First-line use involves topical corticosteroids and oral antihistamines.^3^ In a retrospective cohort study examining treatment outcomes of immune-related cutaneous adverse events, oral antihistamines showed benefit in 81% of patients treated for pruritus.^15^ Efficacy for antihistamines for oncologic pruritus may be limited for non-histaminergic pathways; however, they can provide benefit through sedative properties, especially in patients with nocturnal pruritus.^16^ If the response is unsatisfactory, escalation to immunomodulatory agents is recommended.^3^

For malignancy-associated or paraneoplastic pruritus, including cutaneous lymphoma, treatment is primarily focused on the underlying disease process, with adjunctive use of topical corticosteroids and oral antihistamines. Pruritus affects 66%-88% of CTCL patients and is driven by malignant Th2-skewed T-cell production of pruritogenic cytokines including IL-4 and IL-13, rendering it poorly responsive to antihistamines.^2^^,^^17^

For pruritus associated with GVHD, no standardized recommendations exist. Pruritus severity in cutaneous GVHD varies by subtype, with atopic dermatitis-like cGVHD tending to be more intensely pruritic than lichen planus-like cGVHD, likely due to greater upregulation of TSLP and peripheral eosinophilia.^7^ General itch directed therapy includes topical corticosteroids, topical calcineurin inhibitors, phototherapy, and systemic antihistamines, as well as systemic steroids, and approved systemic treatments ruxolitinib for aGVHD and ibrutinib, belumosudil, and axatilimab for cGVHD^7^.

Despite a very low adverse event rate, sedation (8.5%) was most frequently reported, warranting monitoring in older patients who may be more sensitive to sedation and gait instability and in those with renal insufficiency due to reduced drug clearance.^18^ Starting at a higher dosage of either gabapentin (>300 mg) or pregabalin (>75 mg) has been associated with higher risk for encephalopathy, a fall, fracture, and hospitalization in older adults with chronic kidney disease.^18^ Sedation/drowsiness, a recognized central nervous system effect of gabapentinoids, occurred in 19 patients (8.5%) in our cohort. However, no patients experienced gait instability, falls, or other motor dysfunction, and none required medical intervention for these events. Although our median dose for efficacy was lower for both agents, it is imperative to renally dose gabapentinoids. Patients should also be educated on sedative and gait instability risks associated with dose escalation.

In conclusion, gabapentinoids are a safe and effective treatment for oncologic pruritus. This study has several limitations. Due to its retrospective nature, there is a potential for selection bias. The rationale for administered gabapentin versus pregabalin was not well documented. We did not systematically capture whether patients had a history of pruritus prior to their oncologic diagnosis, as our data collection focused on characterizing pruritus attributable to their cancer or its treatment. Pruritus grading was only documented at clinical visits which typically occurred every few months, contributing to a prolonged median time to CTCAE grade reduction observed; nursing notes and patient-initiated communications confirmed interim improvement between clinic visits. Unavailable data and inconsistencies in EMR documentation complicate analysis and interpretation, and a lack of a control group precludes definitive conclusions regarding efficacy. In addition, patients at our tertiary cancer center present with advanced-stage disease, which may predispose our cohort to severe itch compared to other centers, possibly impacting generalizability. Finally, the high rate of concurrent multimodal anti-pruritic therapy in this cohort limits our ability to attribute observed pruritus improvement to gabapentinoids in isolation.

## Data Availability

The data underlying this article are not publicly available due to patient privacy restrictions. De-identified data may be made available upon reasonable request to the corresponding author, subject to institutional review board approval.
